# RiverCore: IoT Device for River Water Level Monitoring over Cellular Communications

**DOI:** 10.3390/s19010127

**Published:** 2019-01-02

**Authors:** Carlos Moreno, Raúl Aquino, José Ibarreche, Ismael Pérez, Esli Castellanos, Elisa Álvarez, Raúl Rentería, Luis Anguiano, Arthur Edwards, Paul Lepper, Robert M. Edwards, Ben Clark

**Affiliations:** 1 Faculty of Telematics, University of Colima, 333 University Avenue, C.P. 28045 Colima, Col., Mexico; aquinor@ucol.mx (R.A.); jibarreche@ucol.mx (J.I.); ismael_perez@ucol.mx (I.P.); esli_castellanos@ucol.mx (E.C.); arted@ucol.mx (A.E.); 2 Corporativo STR S.A. de C.V., 111-B Canario Street, C.P. 28017 Colima, Col., Mexico; elisa.alvarez@corporativostr.com; 3 Siteldi Solutions S.A. de C.V., 111-A Canario Street, C.P. 28017 Colima, Col., Mexico; raul.renteria@siteldisolutions.com (R.R.); luis.anguiano@siteldisolutions.com (L.A.); 4 School of Mechanical, Electrical and Manufacturing Engineering, Loughborough University, Wolfson Building, Ashby Rd, Loughborough LE11 3TU, UK; P.A.Lepper@lboro.ac.uk (P.L.); R.M.Edwards@lboro.ac.uk (R.M.E.); B.Clark@lboro.ac.uk (B.C.)

**Keywords:** IoT, telemetry, cellular communication, water monitoring, floods

## Abstract

Flooding is one of the most frequent and costly natural disasters affecting mankind. However, implementing Internet of Things (IoT) technology to monitor river behavior may help mitigate or prevent future disasters. This article outlines the hardware development of an IoT system (RiverCore) and defines an application scenario in a specific hydrological region of the state of Colima (Mexico), highlighting the characteristics of data acquisition and data processing used. Both fixed position and moving drifter node systems are described along with web-based data acquisition platform developments integrated with IoT techniques to retrieve data through 3G cellular networks. The developed architecture uses the Message Queuing Telemetry Transport (MQTT) protocol, along with encryption and security mechanisms, to send real-time data packages from fixed nodes to a server that stores retrieved data in a non-relational database. From this, data can be accessed and displayed through different customizable queries and graphical representations, allowing future use in flood analysis and prediction systems. All of these features are presented along with graphical evidence of the deployment of the different devices and of several cellular communication and on-site data acquisition tests.

## 1. Introduction

Floods are among the most frequent natural disasters, causing significant damage to infrastructure and displacing, injuring or killing large numbers of persons. Several studies [1,2,3] have proposed flood warning and prediction systems, implementing Internet of Things (IoT)-based technology to monitor river behaviour before and during flood events to control flood-prone areas, helping mitigate or prevent future disasters.

In 2017, Lamichhane and Sharma [1] state that floods take the lives of more people than many other natural disasters, due to their frequent occurrence and rather unpredictable nature. In the U.S., for example, flash floods kill more than 140 persons per year. The erosive properties of moving water can rapidly debilitate the foundations of structures like bridges and houses that can then be carried off, adding to fatalities and causing billions of dollars’ worth of damage. Between 1980 and 2015, weather-related events like floods accounted for over $500 billion USD in damage in Europe alone [4]. The United Nations Office for Disaster Risk Reduction (UNISDR) consider floods as a key hazard for the regions of Africa, the Arab States, the Asia-Pacific, the Americas, and Europe. In 2016, floods caused 1200 deaths in Bangladesh, India and Nepal. In the same year, sudden-onset natural hazards, primarily floods, caused more than 600,000 new displacements in Africa [5]. This trend is predicted to increase dramatically in the following decades due to climatic change.

Several approaches have been proposed to monitor and provide real-time data of potential flooding using complex sensor datasets and river volume profiles, along with probabilistic methods to predict and warn people of potential disasters.

Rivers, although the most common source of flooding, only represent one potential flooding hazard. Along the western rim of the Pacific Ocean, on average, 20 typhoons per year provide volumes of water that flood canyons and rivers and can accumulate in river basins or deltas where important settlements can be found [2].

The use of IoT technology, along with data analysis, can help monitor floods and provide useful information needed to predict future floods; however, this technology requires significant data processing resources due to large amounts of incoming data, which can result in important delays when measuring real-time scenarios. In order to develop an effective flood monitoring system, IoT technology may be used along with sensors and other technologies, such as machine learning and artificial intelligence techniques, to improve data acquisition and real-time measurement. Also, ultrasonic sensors and cellular transmission technologies can be deployed to ensure adequate transmission rates and to prevent data loss by implementing optimized telemetry methods and lightweight data structures to reduce the load of outcoming data.

Similarly to most natural disasters, floods possess many variables that can be measured by sensors and electronic devices. In the case of floods, these variables usually include flow, volume, speed and relative humidity, among others, as these variables can significantly change how specific environments react under increased water discharge. However, despite being able to measure the different variables associated with floods, it is currently difficult to retrieve real-time water data that can be used to create future forecasting models, due to topographical factors and infrastructure constructed near cities and settlements. This lack of certainty endangers people, property and animals who live near rivers and other bodies of water, particularly in fast-draining areas prone to flash flooding and in low-lying areas such as floodplains and coastal areas. Thus, the main goal of this research is to design, develop and implement a system, based on different electronic devices and technologies, that can measure and monitor potential flooding in real time and generate data that can be used in future forecasting and prediction models.

As more devices access the Internet, more physical objects or “things” can be connected to the cloud, which has created a revolution insofar as data collection is concerned because of the incredible growth in the number of endpoints that collect information users need to view in real time. Experts forecast that by 2020 a total of 50 billion devices will be connected to the Internet [6].

IoT technology provides solutions that improve quality of life by making life easier, safer and more comfortable because of the great variety of application areas in urban, domestic, industrial, agricultural, healthcare, transportation and public safety settings. One important application of this technology relates to efforts to mitigate the impact of natural disasters like floods. In [7] the authors provide a state-of-the-art flood model that can be used to select a suitable method to solve a specific flood problem and discuss some technologies that can be used to remotely sense river data.

The concept of deploying sensors to monitor rivers and streams has been widely tested. Kruger et al. [8] provide the results of several years’ work in which they developed and deployed more than 220 units to monitor water levels. The units employed incorporated an ultrasonic distance measuring module, a solar energy system and a GPS receiver. They also incorporated cell modems to make possible the transmission of data to the Internet via the cellular network. In this implementation, sensors can make frequent (up to every 5 min) stage measurements, and make data available on the Internet. A key feature of the device is its ability to be installed on bridges, an important design feature as bridges provide sturdy mounting platforms and more convenient river access. Finally, to monitor the proper operation of the unit, it incorporates a humidity and a temperature sensor inside the housing. Ultrasonic sensors use sound waves for its ranging and the speed of sound is influenced by several environmental parameters like temperature and humidity. In this case, the temperature sensor also enables the system to improve the accuracy of the ultrasonic sensor.

Lo et al. [9] discuss a series of three measurements of river state using pressure sensors, bubble gauges and float gauges. These sensors potentially demand higher levels of maintenance and could be damaged or destroyed by environmental conditions. They also describe a fourth measurement using non-contact radar gauges. With these sensor types measurement reliability depends directly on variables like humidity, rain and fog; they are also potentially relatively expensive.

There are several methods to sense levels, speeds or distances in rivers or other flood situations, one of which uses ultrasonic waves. An ultrasonic sensing system does not require actual contact with the stream to gather information. It transmits a short burst of ultrasonic waves and acquires a return signal [10]. Using a non-contact system is convenient because it can improve the longevity of the hardware system by not being directly exposed to the constant degradation caused by water and materials that may be carried by stream currents. Additionally, in more sensitive applications, non-contact systems will not contaminate the sensing target as they are not in actual contact with it.

A variety of approaches can be used, some of which can even combine multiple sensors. In [11], the authors use a flash flood sensor combining ultrasonic range finders with passive infrared temperature sensors. This combination of sensors is necessary due to the relationship between the speed of sound and the ambient temperature. It is necessary to estimate and apply a correction to the raw ultrasonic sensor data in relation to air temperature.

Another development that uses multiple sensors is that of Duraibabu et al. [12], which describes in detail the creation of an optical fibre pressure and temperature sensor (OFTPS) to measure these two variables from the same point in either fresh or ocean water. The OFTPS is made of glass and combines a Fabry-Perot interferometer (FPI) to measure pressures and a fibre Bragg grating (FBG) to detect temperatures simultaneously. This sensor is stable due to the material from which it is made, and it performs comparably to the commercial Sea-Bird Scientific SBE9Plus sensor, which makes it commercially competitive.

The set of sensors, communication devices and data acquisition methods, along with telemetry techniques, make the system capable of retrieving useful structured information that can be used in future data analysis and forecasting models. Neal et al. have proposed a forecasting model which uses a network of wireless sensors to monitor the water level of a river for further processing using a one-dimensional hydrodynamic model [13]. The presented data in their research uses variables such as elevation and position of the related nodes along with a Kalman filter to generate data sets as inputs to forecasting models. 

## 2. State of the Art

### 2.1. Sensing High-Speed Water Flows

Electronic devices used to determine water flow measure several variables such as composition, speed and depth, among others. In this context [14] offers nine parameters, which should be taken into consideration to measure a high-speed type of flow (debris flow). Of these, we have chosen to focus on the following:

- Peak flow depth
- Underground sound
- Mean flow velocity
- Surface velocity
- Flow depth as a function of time
- Ground vibration
- Basal forces
- Fluid pore pressure
- Impact force

To analyse these four parameters, they consider using ultrasonic and radar sensors, accelerometers, Doppler speedometers and string potentiometers.

RiverCore’s development will be based on this approach by proposing a combination of these sensors to measure the variables needed to predict flooding and high-speed water flows, achieved by sending real-time analogue and digital data over the cellular network, through the Message Queuing Telemetry Transport (MQTT) protocol, to the Internet.

### 2.2. Distance Sensors and Surface Mapping

According to [7], distance sensors can be used to monitor the Earth’s surface by measuring the distance between a source and its target, using a pulsed laser that returns the distance to the sensor. Distance sensors can generate a three-dimensional matrix by measuring specific points of a defined surface and varying the angle of these measurements. By analysing these sensor-generated data, it is possible to detect increased water levels and determine variations in the flow’s volume.

In order to make projections, the proposed data-logging device must measure the flow’s rise. To do this, it employs an ultrasonic water level sensor to accurately make flood projections over time.

A distance sensor can be used in many applications, including real-time motion tracking for indoor moving spherical objects [15] and being employed as a detection sensor used as a radar to identify fruits or assisting a robot to navigate.

### 2.3. Real-Time Sensor Networks for Data Acquisition

Many hydrological monitoring techniques use real-time data acquisition systems coupled with different electronic devices and sensors to collect more reliable flood prediction information along with hazard warning applications. In this sense, [16] proposes a data-logger device that uses multiple communication protocols to interact with sensors such as the SDI-12 or RS-485 and several analogue inputs. This device is based on a PIC18F8722 low-power 8-bit microcontroller unit. On the other hand, the RiverCore data-logger we propose in this paper is designed using a 32-bit architecture and allows users to connect to a wide variety of sensors which implement the same communication protocols. This 32-bit architecture allows the device to send real-time data from the sensors to the server, and to process incoming signals simultaneously faster than an 8-bit microcontroller. In terms of power consumption, although a 32-bit device will require more energy to operate, it can be improved by designing a low-power routine which keeps the device in sleep mode while not needed and to transmit at a certain moment.

### 2.4. IoT Technologies

There are many technologies used to interconnect devices and to enable them to send collected data to the Internet. Table 1 describes some IoT technologies, their features and their best uses, based on [17,18] for Wireless Wide Area Networks (WWAN), which are divided into Cellular and Low-Power Wide-Area Networks (LPWAN).

### 2.5. Non-Relational (Non-Structured Query Language or NoSQL) Database Systems

IoT devices are gaining popularity due to their ability to gather data and generate information about anything. The large amounts of data generated by this technology, however, need to be stored in some way. IoT has special storage requirements in terms of flexibility, as a large number of devices in a sensor network may not be configured in the same manner and consequently forward different types of data. Another important issue is the availability of all of the network sensors, which is not ensured at all times. 

Traditional database systems implement four transaction properties to maintain data integrity: atomicity, consistency, isolation and durability (ACID) [19]. These four properties ensure data integrity but at the cost of making the database non-adaptable after its initial configuration.

Non-relational databases provide an adequate alternative storage technology because of their ability to store large amounts of data without requiring the strong consistency requirements which typical database systems demand [20]. These types of databases can accommodate multiple data types over time when necessary and even become tolerant of network partitions, thereby providing the possibility of adding multiple storage servers [21,22]. Its ability to accommodate different data types and structures, as well as its scalability and overall flexibility, makes this type of database system a good alternative to meet the storage requirements of IoT networks.

## 3. Materials and Methods

### 3.1. Message Queuing Telemetry Transport

MQTT is an open machine-to-machine connectivity protocol specifically designed to implement IoT solutions, with low data and bandwidth consumption. MQTT is ideal for devices which use low power technology and have small data forwarding requirements [23]. RiverCore is designed to use an MQTT protocol to send data through a SIM cellular communication module which is integrated into its main board. The device sends data strings using AT-Commands from the Microcontroller Unit (MCU) to the SIM module to communicate with an external MQTT broker.

### 3.2. Eclipse Mosquitto™ MQTT Broker

Eclipse Mosquitto™ is an open source-messaging broker that interacts with many devices using the MQTT protocol, which possesses a low code footprint. This broker is installed on a server-side environment that manages all subscription and production topics. The MQTT protocol implements one principal structure, called “topic”, in which messages are sent [24].

The RiverCore server-side environment implements a data acquisition method that uses the Mosquitto broker to receive messages from RiverCore fixed and mobile nodes. These messages are stored in a NoSQL MongoDB database environment.

Given the joint use of the MQTT protocol and a non-relational database architecture, the RiverCore environment can be implemented in a variety of different clients.

### 3.3. MongoDB Non-Relational Database

MongoDB is a database model based on JavaScript Object Notation (JSON) format documents stored in data collections. Because each document has a different structure, any data log is capable of having different attributes [25]. RiverCore’s server-side environment implements MongoDB to save a device’s readings because there could be different sensors connected to the RiverCore datalogger using different log structures in its database. For example, there could be a device holding a temperature sensor and another device possessing an ultrasonic distance sensor, both of which are RiverCore devices; their database entries would have different attributes and values, however. In relational database architectures, this could pose a substantial problem. Importantly, a MongoDB non-relational environment can handle significantly different documents which different devices generate.

### 3.4. Security Considerations in Telemetry Architecture

Because RiverCore employs a wireless transmission environment, it may be vulnerable to sniffing technologies or external intrusions. Taking these possible security threats into account, the system includes security techniques that are embedded in the transmission protocol, which allows the MQTT protocol to set encrypted server-side credentials for each device and user.

Using this same approach, Singh et al. [26] have proposed a message-based security token contained in the message’s header to provide for a unique type of message reserved with the code ‘0000’, which creates a “spublish” method with an encrypted message payload. This security proposal for the MQTT protocol takes into consideration how to register the different devices by assigning them a unique identity by means of a Universal Resource Identifier.

The security techniques used by the RiverCore environment enable it to set encrypted credentials based on Secure Sockets Layer and Transport Layer Security (SSL/TLS) certificates, using the Mosquitto broker’s built-in pre-shared key encryption (PSK).

Non-relational database security methods have also been taken into account, enabling the system to set member and client x.509 certificate-based connections. These measurements ensure public key infrastructure (PKI) formats that certificate users and devices to establish valid connections with the database, whether they are made directly from devices, the data acquisition platform or other certified clients. Similarly, Okman et al [27] have reported some security issues concerning MongoDB, highlighting the lack of authentication of this database engine while in shared mode. To solve this issue, the RiverCore environment can set different security layers, such as SSL/TLS operating system certificates, secure shell (SSH) server access and authentications, which are included in the protocol, as mentioned at the beginning of this section.

### 3.5. RiverCore Fixed Implementation

**Figure 1:**
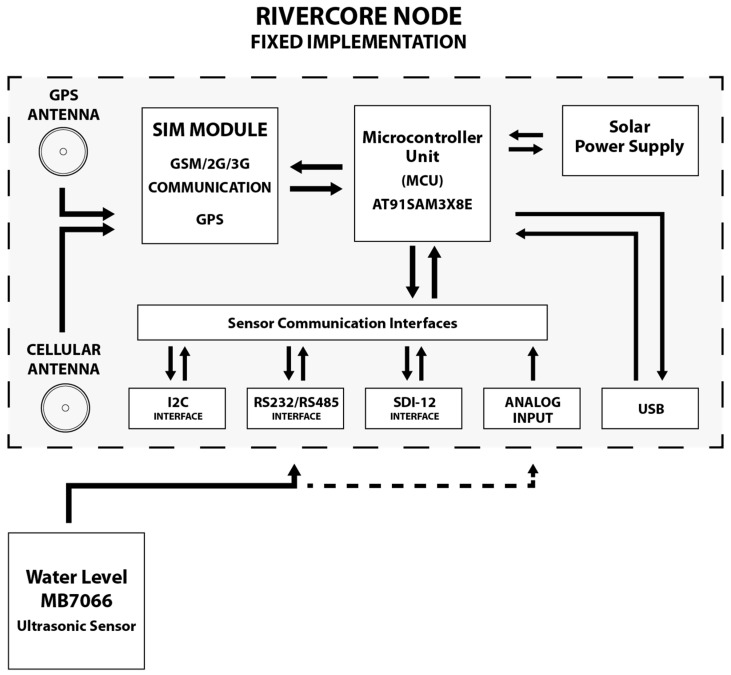
RiverCore fixed node components.

RiverCore is designed to integrate different sensors according to implementation needs. One of these implementations is a fixed RiverCore module that senses different variables in a river, such as width, flow speed and water height by using an ultrasonic distance sensor as shown in Figure 1. RiverCore can send water data through a cellular communications module to monitor variables over a server-side web interface.

**Figure 2:**
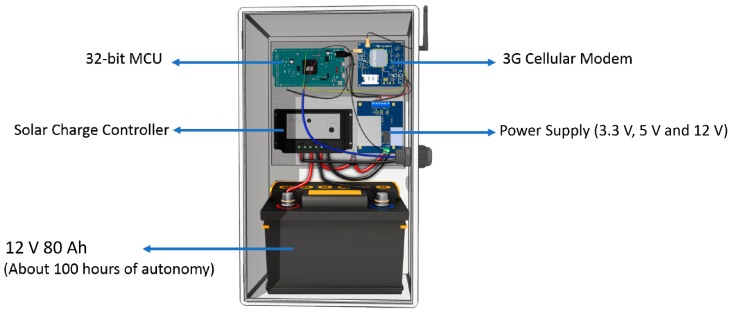
3D model of a RiverCore fixed node.

A RiverCore fixed node is physically composed of five different devices. These are a 32-bit microcontroller unit, a 3G cellular modem electronic board, a regulated power supply, a solar charge controller and a 12 V 80 Ah battery, as shown in Figure 2.

This first prototype of the fixed node contains the proper materials and components to be deployed in a remote location near a river environment to detect water level variations. It is cased in an IP65 enclosure which contains the electronic system along with outdoor designed glands and a battery that allows the system to work continuously during the rainy season without any direct sunlight for four days. This means that the backup battery will allow the device to keep operating when the weather is very cloudy, or during night time, until it can get enough sunlight to recharge. Also, its solar energy management system keeps the battery charged using a 75 W polycrystalline 12 V solar panel which allows the device to recharge before it runs out of energy.

**Figure 3:**
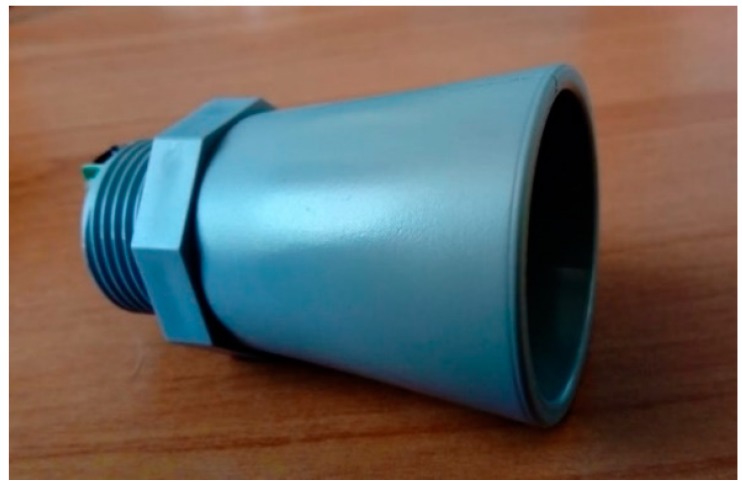
Ultrasonic sensor MaxSonar MB7066.

The ultrasonic water level sensor MB7066 (Figure 3) is used to measure the distance between the water surface and the sensor location, which is processed within the microcontroller and encapsulated in a JSON structure, along with the GPS coordinates of the node, the timestamp of the actual reading and an identification string. This data is then transmitted to the server through the 3G cellular network and stored into the NoSQL database to carry out further calculations that can later be used in a flood forecasting network.

The whole system’s electrical consumption reaches a peak of 1 Ah while transmitting data to the server, using all of its capabilities.

### 3.6. RiverCore Mobile Implementation (Drifter)

**Figure 4:**
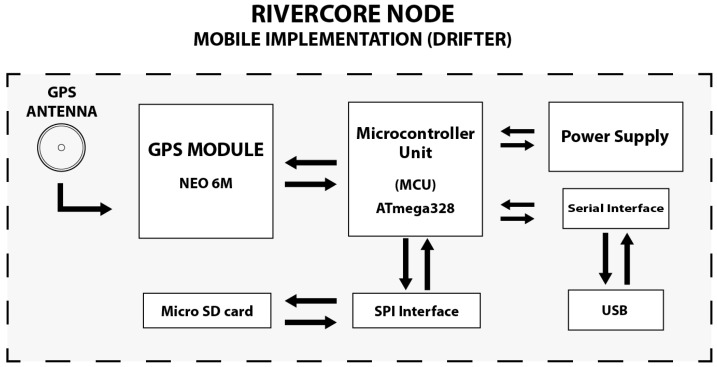
RiverCore mobile node components.

A mobile implementation of a RiverCore node to meet the needs of a monitoring drifter device was also designed. This node integrates a GPS module which retrieves location, time and speed variables. It also contains a micro SD card slot in order to retrieve measured data, as shown in Figure 4. The drifter node is sealed inside a waterproof enclosure that holds a magnetic switch inside, which activates the device to start logging data while it flows through the river bed. This data is stored on the micro SD card and can be analysed by the RiverCore data acquisition platform. No external communication via a cellular or other protocol is built in at this time.

**Figure 5:**
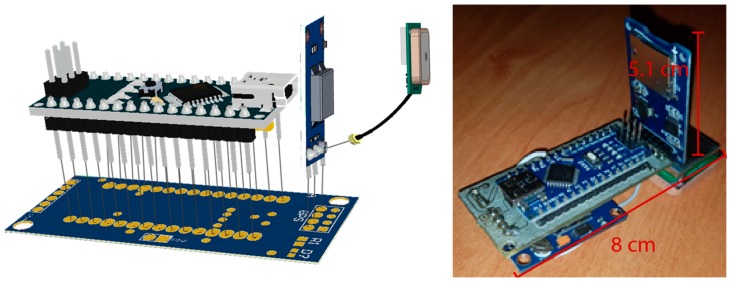
Mobile node 3D model and physical design.

**Figure 6:**
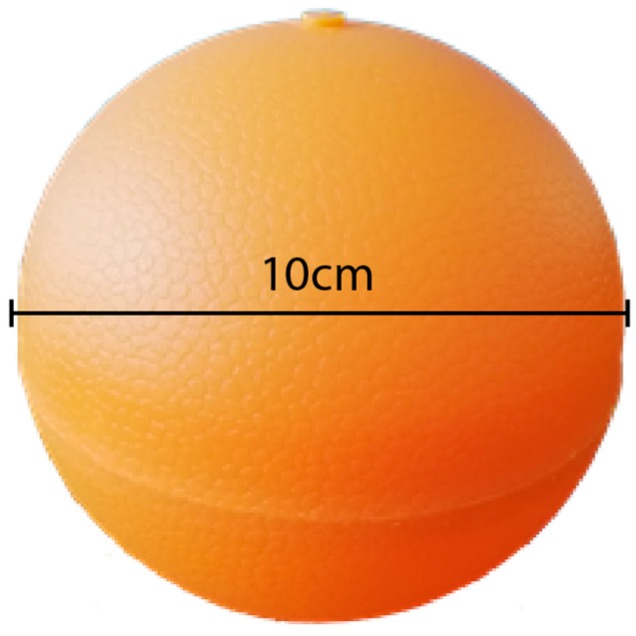
Mobile node lightweight plastic container.

The hardware components depicted above are shown in Figure 5. The electronics were sealed in a spherical lightweight plastic container using a polypropylene rubber-like material that is often used in the automotive industry. This container has a diameter of 10 cm and is shown in Figure 6. The device’s main board is 8 cm long and 5.1 cm high.

The drifting device operates using a magnetic normally-closed switch, allowing it to be turned on after it is sealed by removing a previously attached magnet.

### 3.7. RiverCore Data Acquisition Architecture and Cloud Platform

**Figure 7:**
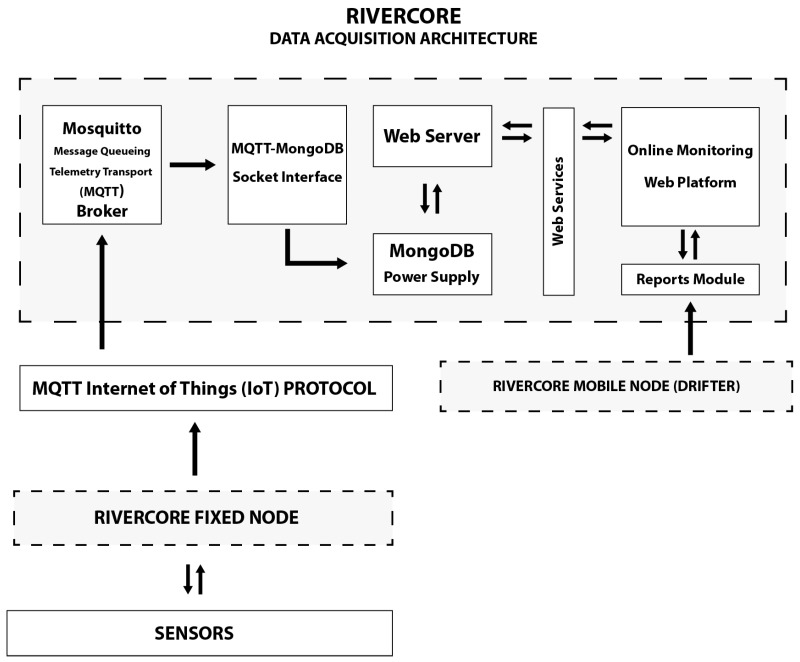
Data acquisition architecture.

As mentioned in previous sections, the RiverCore data acquisition environment is integrated via a hardware device, an MQTT broker and a NoSQL document-based data structure, as shown in Figure 7.

The RiverCore datalogger retrieves information from environmental variables and sends it through a 2G/3G cellular network, using AT commands and the MQTT protocol, to a Fedora 26 Linux server, which has an MQTT Mosquitto broker installed. This broker receives all the messages published to the topic “nodes”, while a background Node.js script saves data using a MongoDB document structure.

While information is saved in a MongoDB database, it can be retrieved using an online monitoring web platform which listens to a WebSocket that receives MQTT messages.

**Figure 8:**
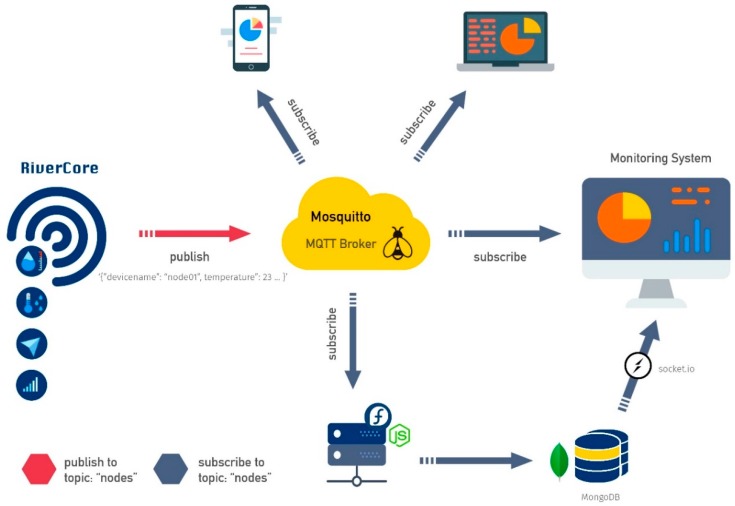
Web platform scheme.

Due to this open scheme, the data acquisition platform is compatible with several devices, software development kits and other IoT projects. By using the JSON format, different devices can publish useful weather data to the system, regardless of the used hardware. Also, devices can subscribe to get useful information in different graphic representations, as depicted in Figure 8.

**Figure 9:**
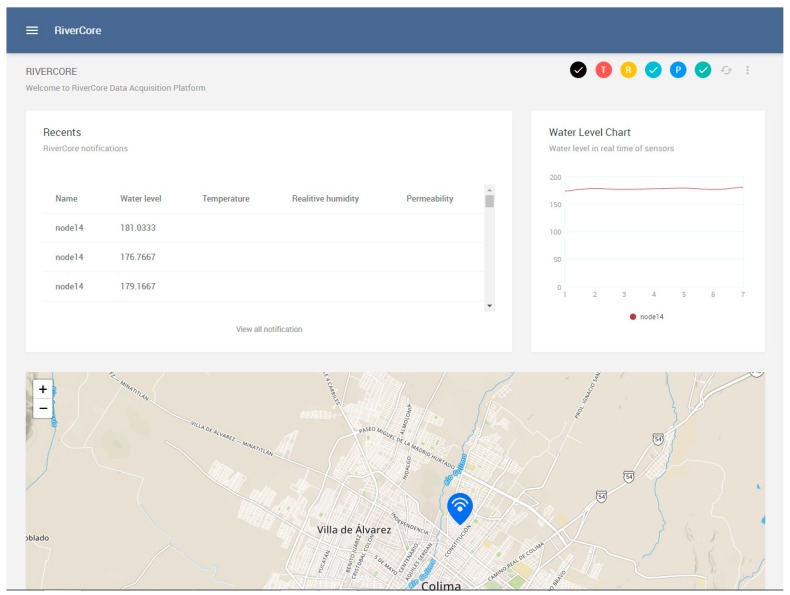
Cloud platform main view.

The web platform integrates a dashboard to display retrieved data from all fixed nodes. This dashboard includes the latest received notifications, variably related charts which it can visualize or hide depending on the sensors that are connected to the devices, and a map where fixed nodes can be located by markers (as shown in Figure 9).

**Figure 10:**
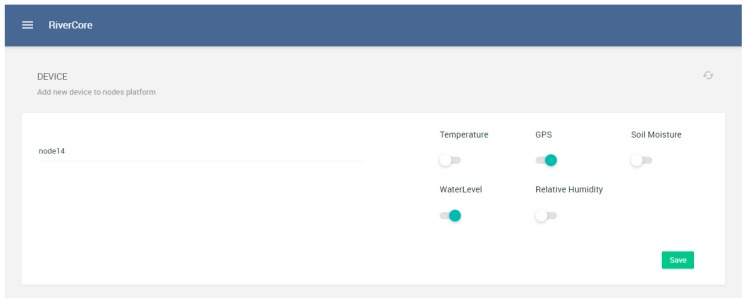
Device registration view.

**Figure 11:**
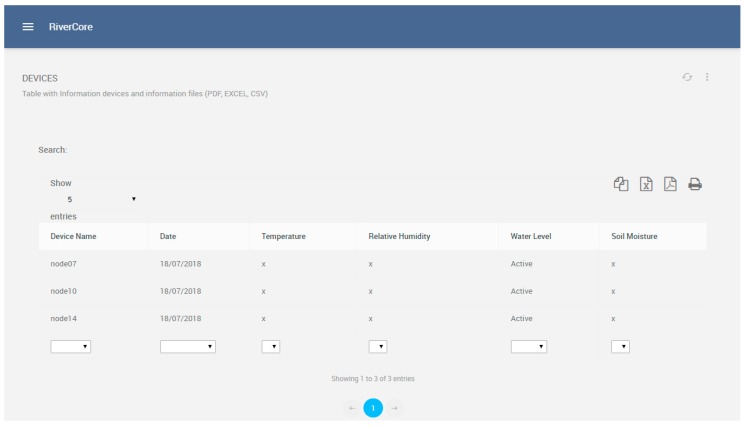
Device management view.

Fixed devices are registered on a dedicated section of the web platform, where they have configuration parameters such as node name and sensor features, as shown in Figure 10. On the other hand, all registered devices can be managed in the section shown in Figure 11, which depicts the features of the registered nodes.

**Figure 12:**
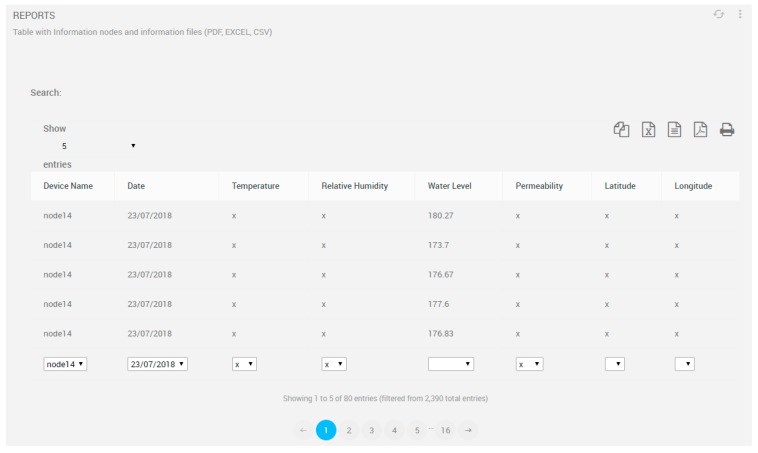
Report exportation view.

Retrieved data from all nodes can be downloaded as reports in several formats such as PDF, EXCEL, directly printed or CSV, and is applicable for further analysis for use in flood forecast systems. Furthermore, data can be filtered by inbuilt queries such as date, node or variable, as is depicted in Figure 12.

## 4. Results

### 4.1. Water Depth, Temperature and Relative Humidity Measurements over Time

Our developed fixed device employs an ultrasonic water level sensor. Additionally, for this trial, we added an environmental temperature sensor and a relative humidity sensor, which we connected to the MCU to send data periodically by means of the MQTT messaging protocol, through the Internet, to the data acquisition server.

**Figure 13:**
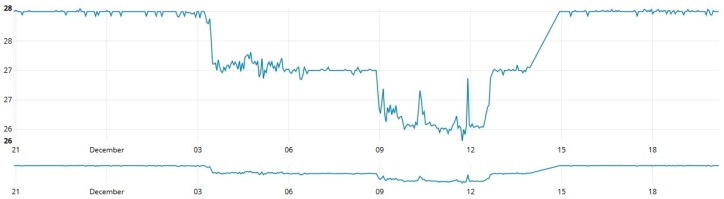
Water depth measurements over time.

**Figure 14:**
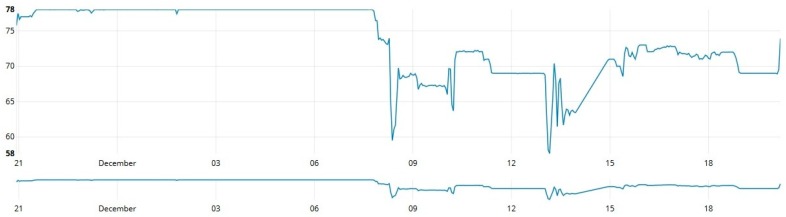
Environmental temperature measurements over time.

**Figure 15:**
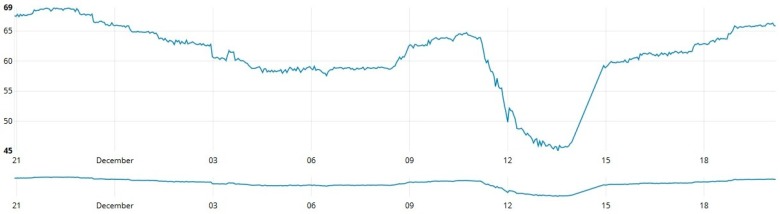
Relative humidity measurements over time.

As shown in Figure 13, Figure 14 and Figure 15, data from these three different sources was collected at one-second intervals during a 24 h journey over a controlled water source. Figure 13 contains data collected by measuring the depth (cm) of the water source, Figure 14 shows the temperature variation during the measurement period and Figure 15 provides a graph that reports the percentage of relative humidity during the period of measurement.

These measurements, transmitted, stored and shown at one-second intervals using RiverCore’s data acquisition environment, demonstrate the reliability of the proposed device in sending real-time data that can predict changes in natural variables.

### 4.2. Fixed Node Deployment and Retrieved Data

In order to deploy the developed fixed node, several 3G signal measurements were made at different locations near the Colima river to determine where to install the device, along with the sensor and the solar panel.

A northern, upriver location was selected where the 3G signal was strong enough to send messages to the data acquisition platform. The location was also selected because water level variances can be detected there, providing a sufficient reaction time before the flow reaches the city of Colima, which is 10 km below the selected point.

**Figure 16:**
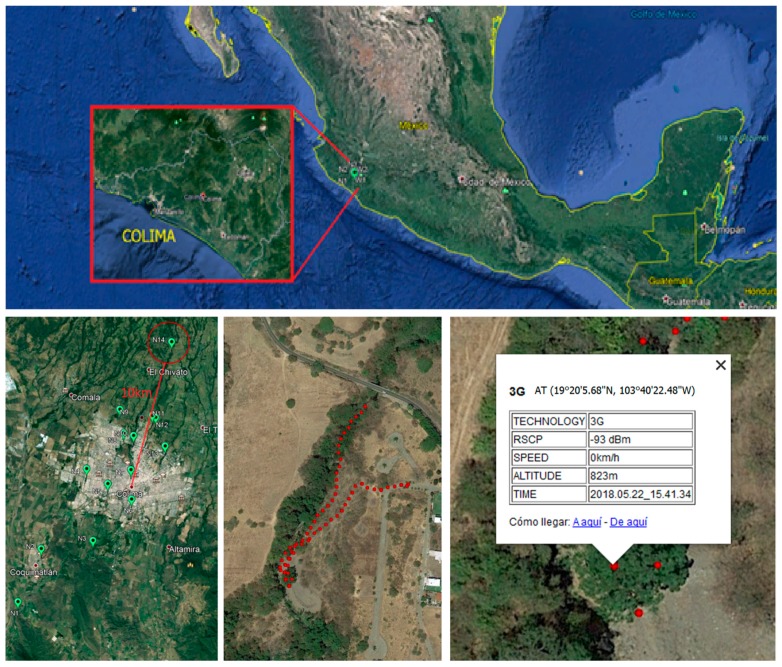
Field work: 3G signal measurements for deployment.

As shown in Figure 16, the complete forecast network is composed of 14 different points along the Colima river or its tributaries. The photograph below is of node number 14, the furthest upriver, which is the first deployed node of the network, where we measured 3G signal strength along the path depicted in Figure 16 (above). Finally, taking signal strength into account, we selected the most reliable location to place the device, which had a Received Signal Code Power (RSCP) value of −93 dBm.

**Figure 17:**
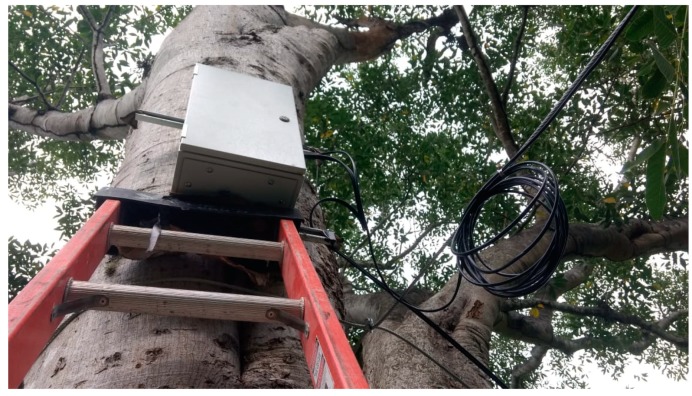
Deployed fixed node.

**Figure 18:**
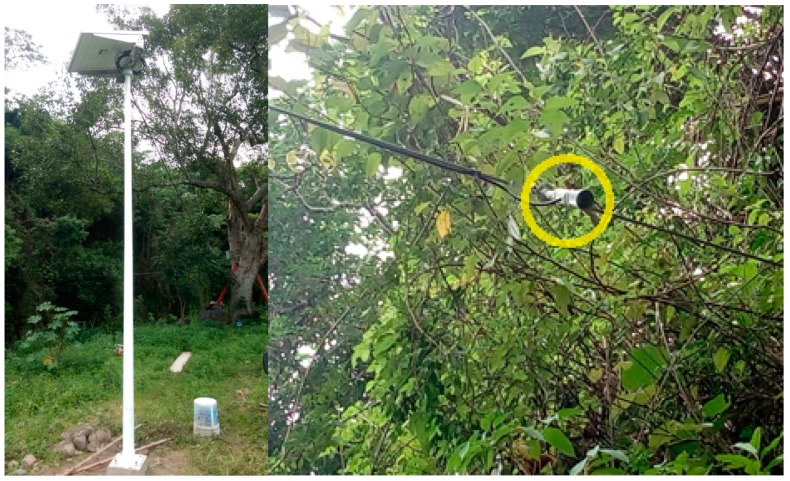
Positioned solar panel and ultrasonic sensor.

The fixed node was attached to a tree at the selected location using stainless steel clamps. From this position, two cables were laid to different parts of the system. The first cable was routed to the polycrystalline solar panel that was mounted on a 4 m high pole, while the second was connected to the MB7066 Ultrasonic sensor, which was firmly fixed to the cliff with a steel wire at a 6 m distance from the IP65 Nema box. The deployment site can be seen in Figure 17 and Figure 18, where the housing containing the electronics and other parts of the infrastructure can be observed.

**Figure 19:**
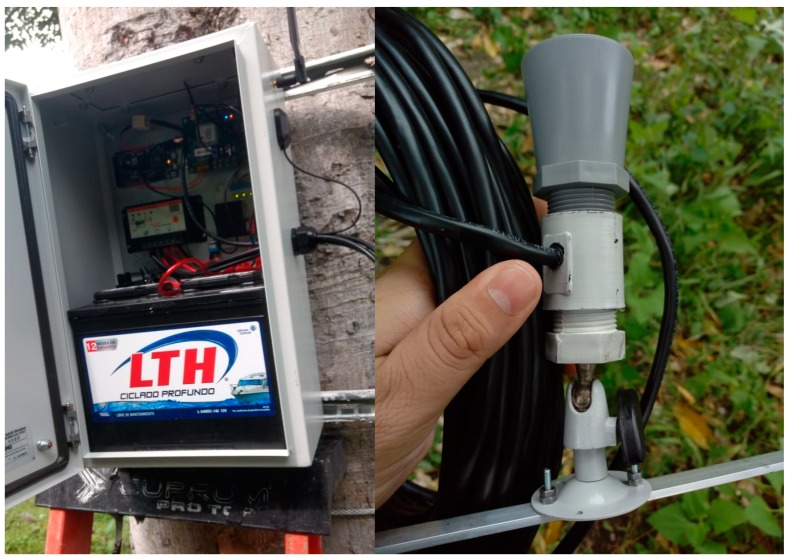
Electronic boards and ultrasonic sensor.

The housing contained the battery and electronic boards related to processing, communication and energy storage of the system. The MB7066 ultrasonic sensor also had an IP67 PVC casing that was mounted in a PVC support along with a stainless-steel adjustable assembly and was connected to the processing unit using a Shielded Twisted Pair (STP) cable as shown in Figure 19.

**Figure 20:**
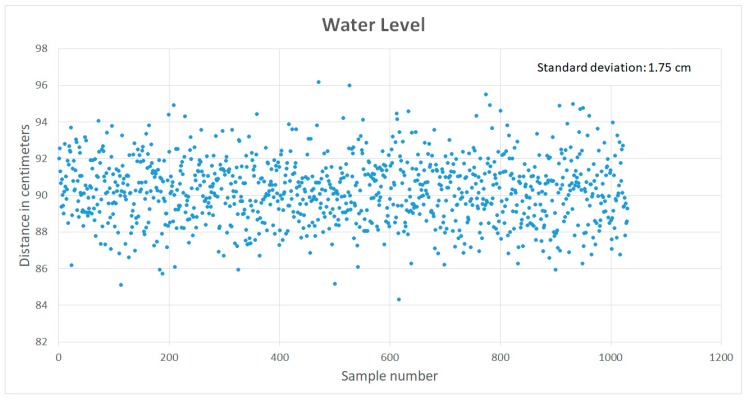
Retrieved data from a deployed node.

After the deployment of the fixed node, measurements were taken to evaluate the operation and performance of the sensor system, the data acquisition platform and the communication platforms. As seen in Figure 20, data was retrieved after 1024 min of normal operation, during which 1024 water level samples had been recorded, at a rate of one sample per minute. During this period, the server registered a standard deviation of ±1.75 cm in the water level of a calm stream. Data was transmitted using a 3G cellular connection through the MQTT communication protocol to the data acquisition platform installed on a Fedora Linux Server.

### 4.3. Mobile Nodes (Drifter) Trials

**Figure 21:**
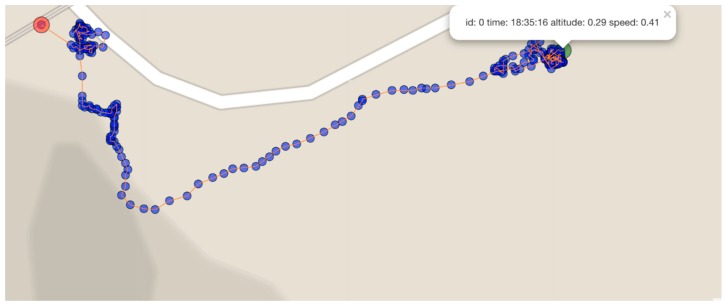
Mobile node GPS sample retrieval.

Trials to evaluate the developed GPS-based mobile (drifter) nodes in a real river environment in order to record their behavior and accuracy while recording GPS positions used in post analysis to determine river flow rates were also carried out. Data of a single downstream drifter GPS position track from trials carried out in a relatively fast flowing river in Southern Colima on 19 July 2018 are shown in Figure 21, where the mobile node was released upstream to record 280 GPS position samples during its operation. The drift was from right to left in the image with a total travel distance of around 194 m. These samples contain data about the traveled path using GPS coordinates. GPS positional data was then used to calculate average speed and distance traveled, and to calculate the river’s slope, among other useful data.

**Figure 22:**
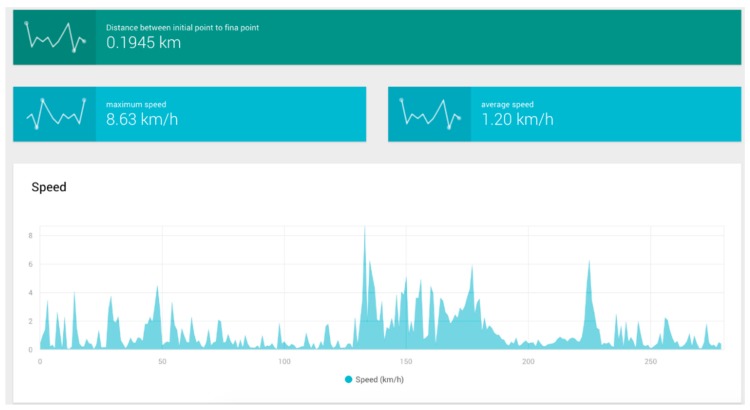
Mobile node speed-related data.

**Figure 23:**
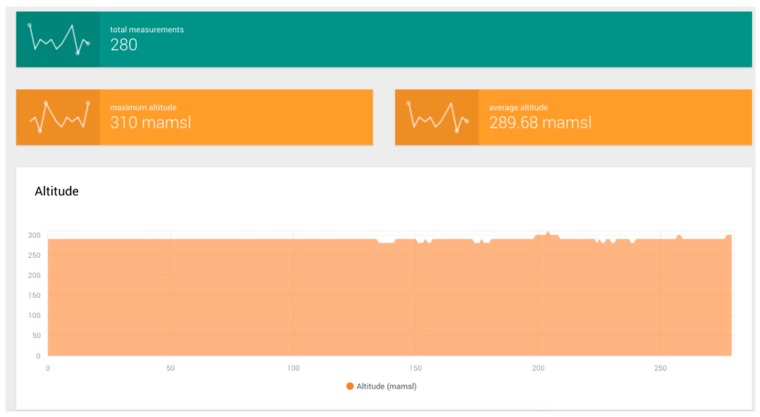
Mobile node altitude-related data.

The drifter trials data were retrieved with the developed data acquisition platform, using as an input a JSON file generated using drifting data along the device’s path. Examples of collected data analysis charts and reports generated from the JSON data format of the drifter data are shown in Figure 22 and Figure 23.

In conjunction with the data acquisition and data rate trials, drifter nodes were also deployed along the river’s path near the fixed node locations. The data obtained from these activities is shown in Table 2; it corresponds to samples measured in different parts of the Colima river which represent different flow conditions and environments such as those that are rocky, those with high levels of vegetation and those with other obstacles that hinder the drifter’s trajectory. 

The total number of samples, speed and distance traveled varies depending on whether the drifter can or cannot avoid certain objects during its path. Therefore, a bigger number of samples does not directly correspond to a longer distance nor to a faster trajectory but can refer to a bigger quantity of obstacles. The total distance measured during our total set of trials was 2655.74 m.

## 5. Conclusions and Discussion

Upon developing, testing and implementing the RiverCore IoT devices and RiverCore’s data acquisition environment, we found three important aspects that require further discussion.

The first important point is the acquisition rate. While developing this real-time device, it was important to measure and store variables using the minimal amount of time possible to permit time to establish communications with the Internet connection between the hardware and the data acquisition web platform. This should be sufficiently low to minimize any significant loss of data that could compromise the system’s ability to monitor the river’s water level. It is also important to remark that the system, as a whole, should ideally be composed of several fixed nodes in order to ensure the operation of a network as a source of useful information for forecasting models, in line with the one mentioned in [13]. However, the present single prototype functions as a proof of concept useful in evaluating the behaviour of single nodes in realistic field conditions which allow later scaling to multi-node networks.

Secondly, 2G/3G cellular networks communication signals can suffer from interference and strong signal variances in remote locations, increasing the possibility of losing important data. A solution for this common problem is to develop mechanisms like data pools or data files that store most of these data in the device’s memory so that if the Internet connection is lost, the data can be sent at a more suitable moment. By implementing the same storage device that is used in mobile nodes, it is possible to keep data during connection losses.

Similarly, it would be useful to add a real-time connection to mobile drifter nodes to track their location after storm conditions or in case of sensor loss. In this case it may be dangerous to retrieve them if water levels have not sufficiently dropped or the stream flow is too rapid.

Other planned future improvements include enhancing RiverCore fixed- and mobile-node capabilities by making them compatible with commercially available weather stations using the SDI-12 protocol, and establishing mixed interactions between drifters and fixed devices to ensure more efficient data transmission to web-based data acquisition platforms. In terms of power efficiency, future improvements for fixed-node devices include implementing sleep and stand-by modes in order to enhance battery duration and power supply performance while not in the rainy season.

It is important to mention that 4G or 5G network signal strength is not adequate in more remote areas in Mexico, with 3G coverage currently offering better reception for IoT devices. However, our testing has shown that there is no need for a faster connection or more bandwidth in this particular application. This is due to the use of a small data packet and a light communication protocol.

However, the examples of data retrieved from sensor devices deployed in remote and/or urban river locations demonstrated in this paper could be used in forecasting models. Close consultation with hydrologists and data scientists will be needed to implement machine-learning algorithms and other forecasting methods for use in a more extended network formed by many of these devices, along with the implementation of a real-time warning method based on a one-dimensional hydrological model, as mentioned in [7], supported with topographic basin surveys to establish secure-prone thresholds in each one of the nodes.

Thanks to the data generated and coordination with the local authorities, we can conclude that a complete Emergency Water Information Network (EWIN) might reduce the impact of floods in our city, providing information for city planning and in a worst-case scenario giving citizens several minutes to get away from flood zones.

New monitoring developments that include IoT technology have led to considerable hazard reduction and better understandings of natural phenomena. Here we have shown that building and deploying a reliable water level monitoring network can be achieved with current technology. Nowadays, cellular networks are well developed over most countries, providing a suitable platform upon which to deploy monitoring networks. This does not take away from the fact that cellular networks can fail during natural disaster situations, as with any other wireless technology. Because most water bodies are not equipped with any sensing technology, despite the risks they represent, this is an important cornerstone for hazards and natural fluid dynamics research. Oftentimes the limiting factor is the cost of each individual sensing node, and what we have shown is that a low cost and reliable water level monitoring network is indeed feasible.

## Figures and Tables

**Table 1:** Review of IoT Technologies.

Name		Frequency	Range	Data Rate	Best Use
Cellular	3G/4G/LTE	900 MHz–2.6 GHz	Depends on area coverage	1 Mbps	For data intensive interactive user services like videoconferencing or telemedicine.
Low-Power Wide-Area Network (LPWAN)	SigFox	Regional sub-GHz bands	3 to 50 km	100 bps up, 600 bps down	For applications that need to send small or infrequent data bursts such as alarm systems or simple metering.
	LoRaWAN		2 to 15 km	0.3 kbps–50 kbps	For long-range and low-power connectivity applications.
	OnRamp/Ingenu	2.4 GHz	500 km	20 Kbps	Offers long-range connectivity for smart grid, intelligent lighting and advanced metering infrastructure, among others.
	Weightless P	Regional sub-GHz bands	Up to 10 km	200 bps to 100 kbps	Ideal for private networks where both uplink data and downlink control are important.

**Table 2:** Summary of drifter retrieved data.

Location (Lat/Lng)	Total Samples	Max Speed	Total Distance
(19°10′26.04″ N), (103°49′45.43″ W)	280	8.63 km/h	194.50 m
(19°10′26.04″ N), (103°49′45.43″ W)	293	8.3 km/h	234.10 m
(19°12′28.07″ N), (103°45′42.06″ W)	556	10.21 km/h	390.05 m
(19°12′28.07″ N), (103°45′42.06″ W)	562	9.42 km/h	401.30 m
(19°15′0.63″ N), (103°43′29.14″ W)	276	6.7 km/h	193.21 m
(19°15′0.63″ N), (103°43′29.14″ W)	302	6.2 km/h	190.32 m
(19°20′5.68″ N), (103°40′22.48″ W)	626	7.14 km/h	533.75 m
(19°20′5.68″ N), (103°40′22.48″ W)	631	6.92 km/h	518.51 m
